# Methane, Ethane, and Propane Detection Using a Quartz-Enhanced Photoacoustic Sensor for Natural Gas Composition Analysis

**DOI:** 10.1021/acs.energyfuels.4c03726

**Published:** 2024-12-19

**Authors:** Aldo F. P. Cantatore, Giansergio Menduni, Andrea Zifarelli, Pietro Patimisco, Marilena Giglio, Miguel Gonzalez, Huseyin R. Seren, Pan Luo, Vincenzo Spagnolo, Angelo Sampaolo

**Affiliations:** † PolySense Lab, Dipartimento Interateneo di Fisica, University and Polytechnic of Bari, Via Amendola 173, Bari 70126, Italy; ‡ PolySense Innovations srl, Via Amendola 173, Bari 70126, Italy; § Aramco Services Company, 17155 Park Row, Houston, Texas 77084, United States; ∥ EXPEC Advanced Research Center, Saudi Aramco, Dhahran 31311, Saudi Arabia

## Abstract

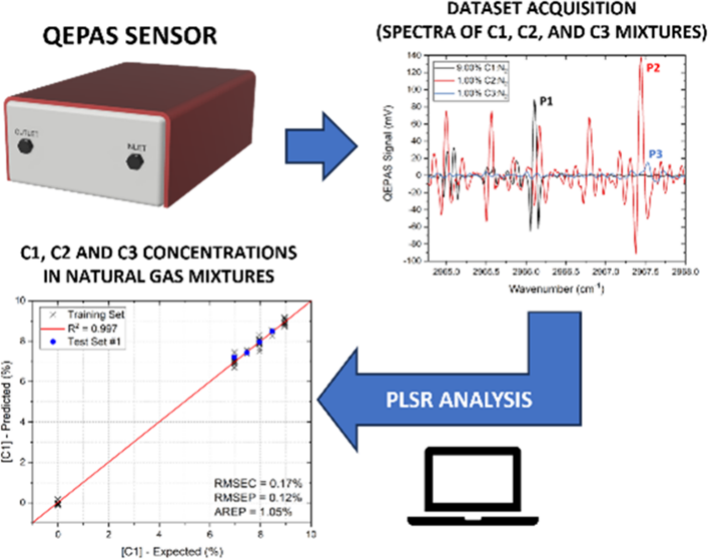

A compact and portable gas sensor based on quartz-enhanced photoacoustic spectroscopy (QEPAS) for the detection of methane (C1), ethane (C2), and propane (C3) in natural gas (NG)-like mixtures is reported. An interband cascade laser (ICL) emitting at 3367 nm is employed to target absorption features of the three alkanes, and partial least-squares regression analysis is employed to filter out spectral interferences and matrix effects characterizing the examined gas mixtures. Spectra of methane, ethane, and propane mixtures diluted in nitrogen are employed to train and test the regression algorithm, achieving a prediction accuracy of ∼98%, ∼96%, and ∼93% on C1, C2, and C3, respectively. With respect to previously reported QEPAS sensors for natural gas analysis, the high prediction accuracy as well as the capability to discriminate and detect C3 within natural gas-like complex mixtures provided by the employment of partial least-squares regression mark significant improvements. Furthermore, these results enable an improved performance of the sensor for in situ, real-time, and online natural gas composition analysis.

## 1 Introduction

Gas detection and compositional analysis are fundamental across the oil and gas industries. During the exploration and production of hydrocarbon fluids, gas may be captured for analysis from downhole tools, the wellhead, from separators, or extracted from drilling fluids at the surface through mud logging operations.^1,2^ The relative abundance of different light hydrocarbon species (from methane to pentane, C1–C5) in a gas sample can be used to determine the properties of the gas (e.g., wetness, density), and their ratios are useful in fluid typing and fingerprinting.^3,4^ Gas composition information also helps in the economic evaluation and designing of the facilities for production.^5^ Once gas plants refine a commercial natural gas (NG) product, its composition is monitored to determine its calorific value, which is related to its combustibility and therefore indicative of its quality and final price.^6^ The calorific value is then monitored during distribution as gas from different sources is blended, transported, and sold to end-users. Up to date, standard methods for the analysis of natural gas composition require the use of gas chromatographs (GCs), frequently coupled to flame ionization detectors (GC-FIDs) or mass spectrometers (GC-MSs).^7,8^ These conventional methods typically require extensive maintenance and calibration tasks in the field. On the other hand, optical spectroscopy techniques, while being simpler, more cost-effective and reliable, and better suited for online monitoring, have been hindered by challenges in obtaining quantitative composition information due to their overlapping spectral components in natural gas samples. However, when combined with chemometric methods, optical spectroscopy can provide improved quantitative predictions.^9^

Despite being characterized by an extremely high sensitivity, GC analysis has multiple drawbacks, which ultimately depend on either the detectors or the hyphenated systems deployed.^8,10^ For example, FIDs display an almost universal response to organic compounds, thus becoming strongly limited when applied to composition analysis.^8^ On the other hand, the main drawbacks of GC-MS systems are represented by (i) the need for high vacuum pumps (down to 10^–7^–10^–6^ Pa), (ii) the coupling interface between the two instruments, (iii) the cross-interferences in the mass spectra, (iv) the analyte separation intrinsic to the technique, and (v) the overall cost of the whole apparatus.^8,9^

Gas sensing systems based on optical spectroscopy have been demonstrated to be a solid and reliable alternative to traditional analytical techniques for hydrocarbon detection.^11−14^ Among the large number of optical-based techniques, gas sensors based on quartz-enhanced photoacoustic spectroscopy (QEPAS) represent a solid alternative to GCs for NG analysis, thanks to their compactness, ruggedness, high selectivity and sensitivity,^15^ as well as low cost and capability of performing online, dynamic flow, and nondestructive measurements.^16,17^ Indeed, QEPAS sensors have already been successfully deployed for the detection of lighter alkanes, i.e., C1, C2, and C3, from traces to high concentrations.^18−23^

QEPAS is an indirect absorption technique, which is developed as a variation of traditional photoacoustic spectroscopy (PAS), employing a quartz tuning fork (QTF) as a sharply resonant transducer. A laser beam, whose wavelength is resonant with an absorption feature of the target molecule, is focused between the QTF prongs and modulated at its resonance frequency or one of its subharmonics. Then, the weak sound waves, generated through the relaxation of the excited molecules via nonradiative energy transfer processes,^17,24^ are detected and converted into an electric signal, exploiting the piezoelectric properties of quartz. As reported in the literature, QEPAS response is dependent on the sample composition, influencing both the photoacoustic relaxation cascade and the sound wave propagation.^25−27^ Fluctuations in the mixture composition may lead to variations of the gas density, influencing both the sound speed and the QTF quality factor.^27,28^ Conversely, the relaxation rate of the excited molecule is affected by the different components of the mixture, having an effect on its relaxation pathway and, in turn, on the QEPAS signal. These effects, known as “matrix effects”,^19,29,30^ together with overlapping spectral features of different analytes, are the most challenging aspects of QEPAS signal processing.^29,31,32^ The issue of spectral overlap is crucial in hydrocarbon detection by optical spectroscopy. In particular, the absorption bands of multiple hydrocarbons fall in the spectral range around 3.3 μm, related to a stretching of the C–H bond common to all of the alkanes.^33^ A first tentative quantification of C1, C2, and C3 concentrations in a diluted natural gas-like gas sample was demonstrated by Luo et al.,^23^ employing a QEPAS sensor with a 3345 nm interband cascade laser (ICL) as a laser source and a standard QTF as a sensitive element. The evaluation of the sample composition relied on a complex algorithm mainly based on univariate calibrations for the three main alkanes and exploiting the saturated absorption of C1 at tens of percent scale to evaluate the effect of the C2 and C3 fluctuations in terms of cross-sensitivities on methane detection. This approach has proved to have limitations in modeling the energy relaxation in multicomponent gas samples, and it does not offer a clear advantage in terms of response time, even though the signal interpretation is based on peak signal extraction rather than extended spectra acquisitions.^23^ Recently, partial least-squares regression (PLSR) has been proposed as a multivariate analysis (MVA) technique to filter out matrix effects and spectral interferences in the QEPAS response, thus providing a more accurate prediction of every single analyte concentration in complex gas mixtures compared to the standard univariate calibration.^19,30,34^

In this work, we report on a compact and portable QEPAS sensor to detect C1, C2, and C3 in NG-like mixtures, employing an ICL emitting at 3.367 μm to exploit the most intense absorption band of propane in the whole infrared region. NG-like mixtures are referred to as mixtures containing C1, C2, and C3 in typical NG concentrations but diluted in a 1:10 ratio in pure nitrogen. PLSR is used to retrieve the analyte concentrations, removing both spectral interferences and matrix effects. The data analysis was performed exploiting a machine-learning-like approach, training the algorithm using C1–C2–C3 mixtures, whose concentrations varied in the range of 7–9%, 0–1%, and 0–1% within a nitrogen matrix, respectively. The obtained results demonstrate the suitability of the developed sensor as a versatile tool for real-time and in situ monitoring of the most significant geochemical fingerprints in natural gas, even with widely variable gas matrices, paving the way toward a fully integrated QEPAS-based NG analyzer system.

## 2 Sensing Architecture for Methane, Ethane, and Propane Detection

**Figure 1:**
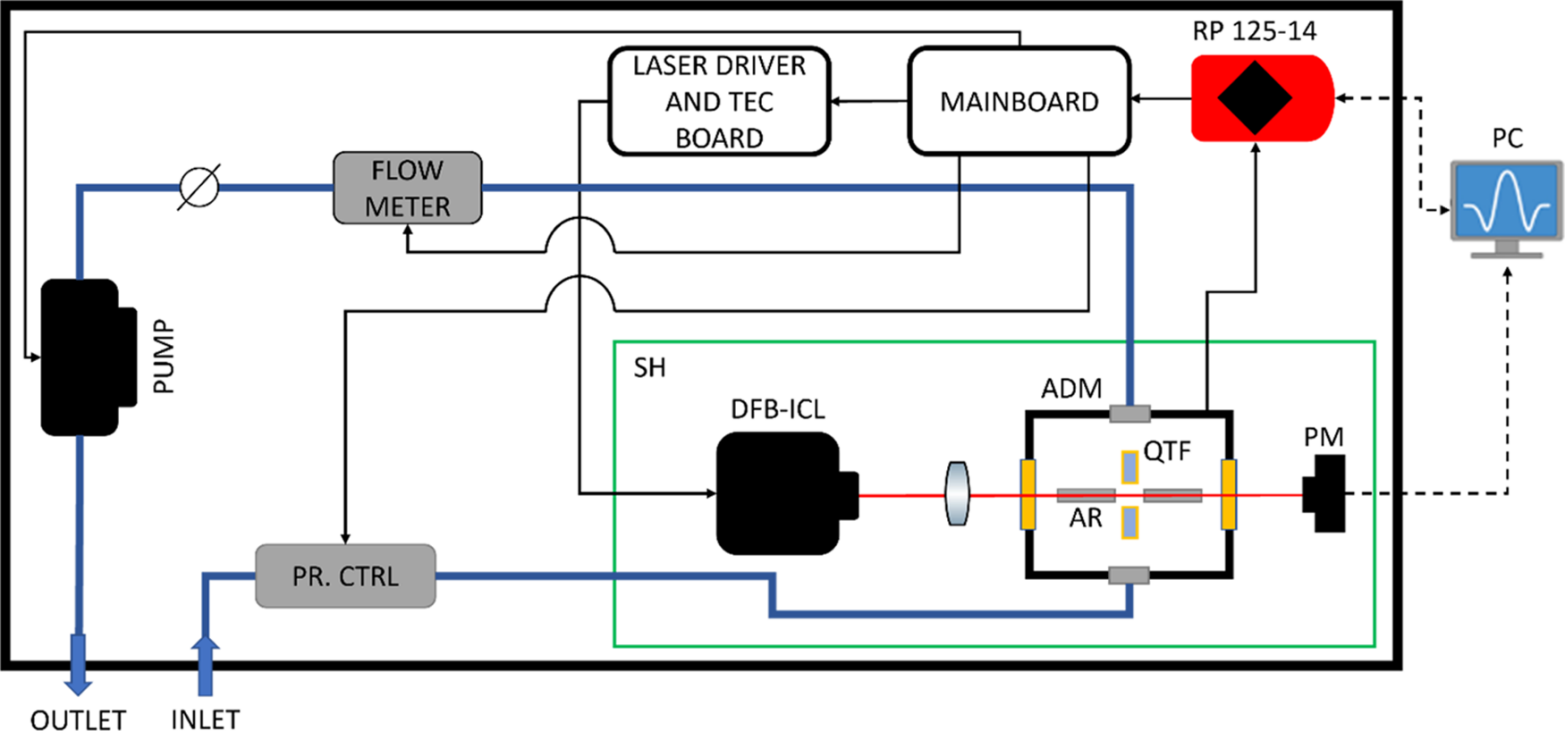
Architecture of the QEPAS sensor. Black arrows represent electronic connections, bold blue lines represent gas handling line connections, and dashed black arrows represent USB connections. The box walls are represented as bold black rectangles. DFB-ICL - Distributed FeedBack Interband Cascade Laser, ADM - Acoustic Detection Module, QTF - Quartz Tuning Fork, AR - Acoustic Resonator, PM - Power Meter, SH - Sensor Head, TEC - ThermoElectric Cooler, PR. CTRL - PRessure ConTRoLler, RP - RedPitaya, and PC - Personal Computer.

A scheme of the QEPAS sensor employed to acquire the spectra associated with mixtures of C1, C2, and C3 in a pure nitrogen matrix is depicted in Figure 1. The sensor is enclosed within a portable 19″-diagonal aluminum rack box.

The sensor head (SH) hosts the optical components of the sensor, preventing possible misalignments, thus increasing its ruggedness. Light emitted by a DFB-ICL (Nanoplus GmbH), with a central emission wavelength of 3367 nm (2970 cm^–1^) and a peak power of ∼11 mW at 23 °C, is focused through an acoustic detection module (ADM, Thorlabs ADM01^35^), i.e., a vacuum-tight gas cell equipped with two ZnSe wedged windows with a 2–13 μm antireflection coating (Thorlabs WG70530-E4) and two connectors for gas inlet and outlet. The ADM encloses a spectrophone consisting of a T-shaped quartz tuning fork (QTF) acoustically coupled to a pair of acoustic resonator (AR) tubes, exhibiting an overall resonance frequency *f*_0_ = 12461.6 Hz and a *Q*-factor ≅ 16,500 at an operating pressure of 200 Torr. The QTF output piezoelectric current is converted into a voltage signal by means of a transimpedance amplifier with a 10 MΩ feedback resistor (not shown in the setup scheme). A power meter (Thorlabs S120C) is placed behind the ADM for alignment purposes, and it is connected to a PC via a USB connection.

The QTF output signal is fed to a RedPitaya (RP) STEMlab 125-14 board, which is also used to control a laser driver and a thermoelectric cooler (TEC) driver chip (Thorlabs MTD1020T), mounted on a dedicated PCB. A mainboard bridges the electronic connections from the PCB to the RP board, controlled by a PC by means of dedicated LabVIEW-based software. All of the measurements were performed in wavelength modulation and second harmonic detection (WM-2f), modulating the laser current sinusoidally at half of the spectrophone resonance frequency and demodulating the spectrophone output signal at the QTF resonance frequency.^36^ A slow ramp can be superimposed to the sinewave to scan the laser dynamic range. Both the modulation and demodulation processes are handled by the RP board and a LabVIEW-based dual-phase digital lock-in amplifier having a maximum input voltage of 1 V.

The measurements were performed at an operating pressure of 200 Torr and a gas flow of 25 sccm. These conditions were kept stable and monitored by means of a pressure controller (Alicat EPC-15PSIA-P0), a flow meter (Axetris MFM 2220-BA-U0), a needle valve, and a diaphragm pump (Thomas 1420BLDC), connected to the RP board through the mainboard. NG-like mixtures of the three alkanes diluted in a nitrogen matrix were generated by means of a gas mixer (MCQ Instruments GB-103). The gaseous samples were generated using cylinders with the following certified concentration: pure C1 (99.50%), 9.85% C1:N_2_, 1.00% C2:N_2_, and 1.00% C3:N_2_, each one characterized by an expanded relative uncertainty of 2%.

## 3 Single Analyte Sensor Calibration and Matrix Effects in Two Gas Mixtures

**Figure 2:**
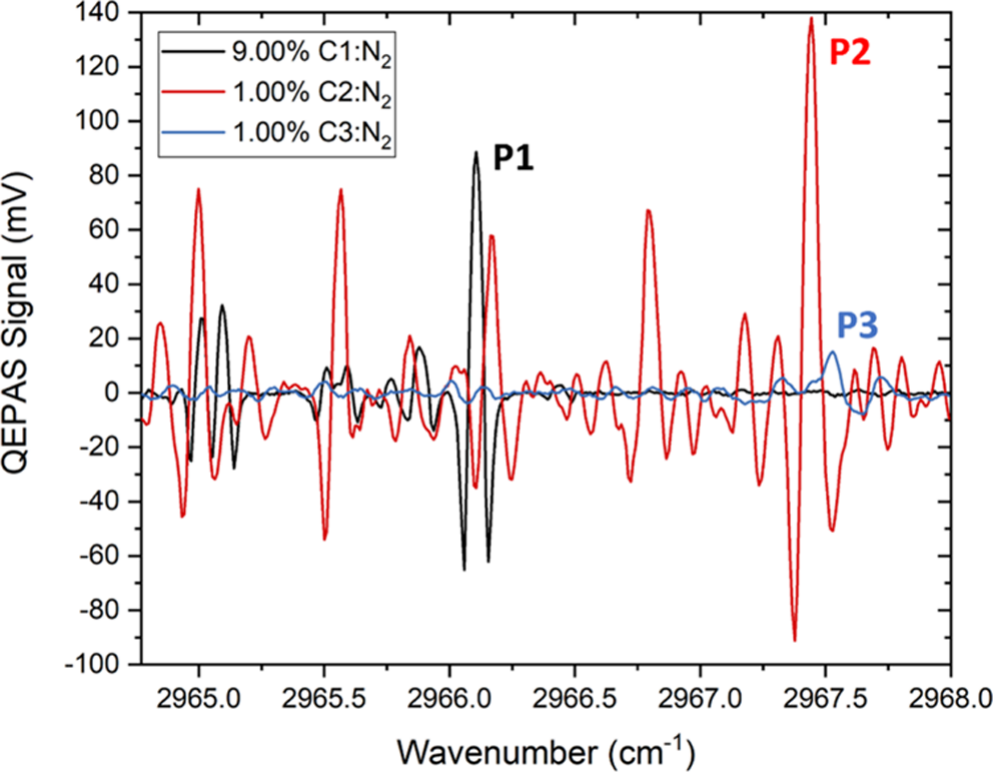
Examples of the single analyte spectra acquired for a 9.00% C1 (black solid line), 1.00% C2 (red solid line), and 1.00% C3 (blue solid line) concentration in nitrogen within the 2964.77–2968.00 cm^–1^ spectral range.

The QEPAS sensor was first calibrated for C1, C2, and C3 separately. The explored C1 concentration range for the calibration spans from 0.99% to 9.85%, while the C2 and C3 concentration ranges span from 0.10% to 1.00%. Performing the measurements at 200 Torr is beneficial since it is a sufficiently low pressure to ensure a sufficient spectral separation among the absorption features of the three analytes.^37^ Examples of the single analyte QEPAS spectra acquired by scanning the laser dynamic range from 2964.77 cm^–1^ to 2968.00 cm^–1^ are shown in Figure 2.

**Figure 3:**
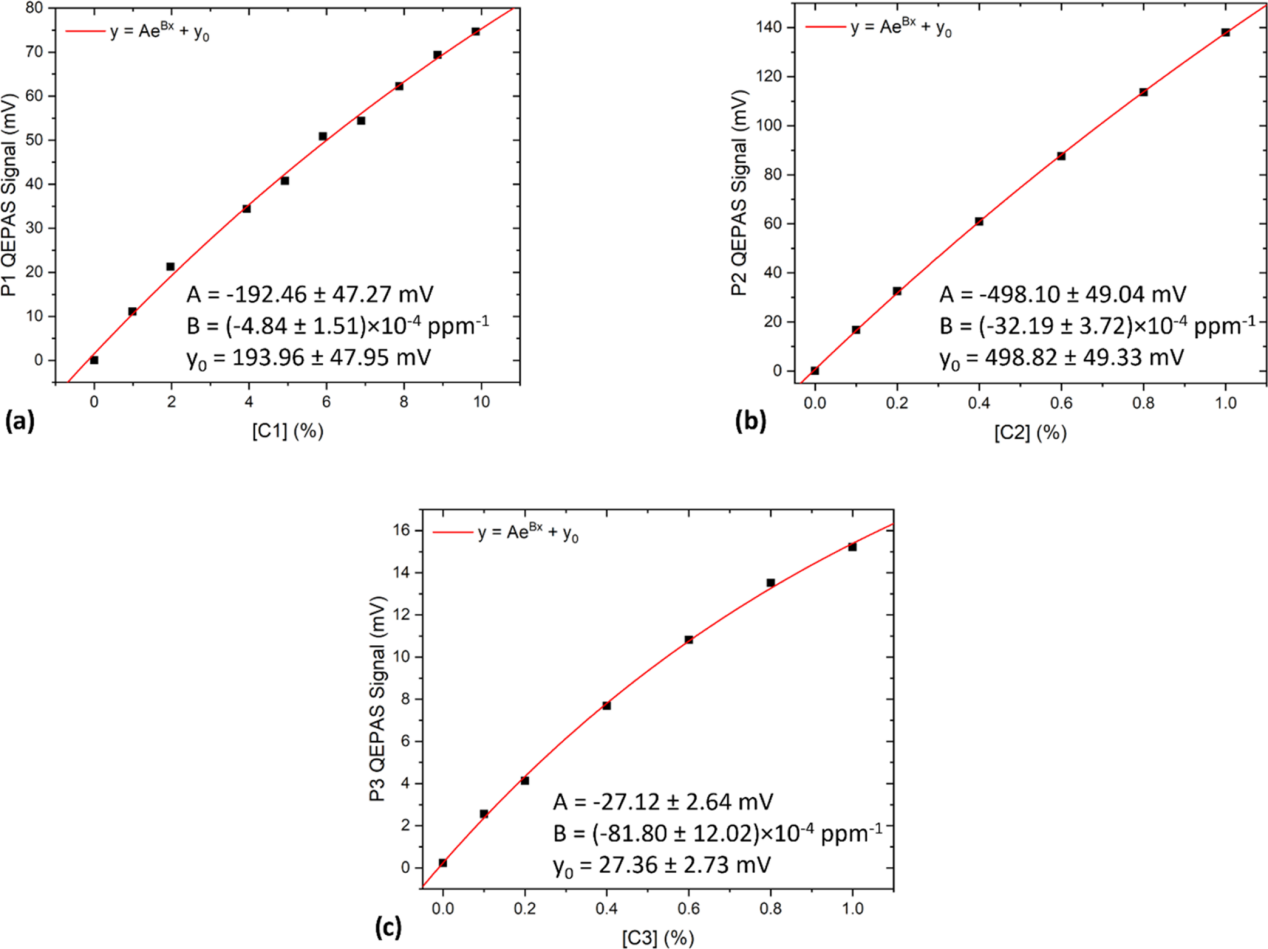
Calibration curves of (a) C1 performed on the P1 peak in the 0.99–9.85% concentration range; (b) C2 performed on the P2 peak in the 0.10–1.00% concentration range; (c) C3 performed on the P3 peak in the 0.10–1.00% concentration range. In each case, an exponential fit (solid red line) is superimposed on the experimental points.

The spectra of the three components exhibit a high overlap in the investigated tuning range. The single analyte calibrations were performed by monitoring the P1, P2, and P3 peaks of C1, C2, and C3, respectively, at different analyte concentrations. The first two peaks correspond to the ro-vibrational transitions falling at 2966.11 cm^–1^ (C1) and 2967.49 cm^–1^ (C2).^37^ P3 is the most intense peak detectable in the targeted spectral range, belonging to a broad C3 absorption band, as reported in the PNNL database.^38^ The calibration curves obtained plotting the QEPAS signal at different C1, C2, and C3 concentrations are shown in Figure 3a–c.

A moderate nonlinear sensor response was observed for all of the analytes, which is consistent with the behavior shown by the QEPAS signal when dealing with high concentrations of the target analytes.^19^ Indeed, when the target analyte concentration is too high to consider the first-order approximation of the Beer–Lambert law, a nonlinear trend is expected.^19^ Therefore, an exponential fit curve was superimposed on the measured data:
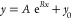
1

The retrieved fit parameters are reported in Table 1.

**Table 1:** Parameters Defining the Exponential Fit Superimposed to the Single Analyte Calibrations Performed on C1, C2, and C3

	*A* (mV)	*B* (10^–4^ ppm^–1^)	*y*_0_ (mV)
C1	–192.46 ± 47.27	–4.84 ± 1.51	193.96 ± 47.95
C2	–498.10 ± 49.04	–32.19 ± 3.72	498.82 ± 49.33
C3	–27.12 ± 2.64	–81.80 ± 12.02	27.36 ± 2.73

**Figure 4:**
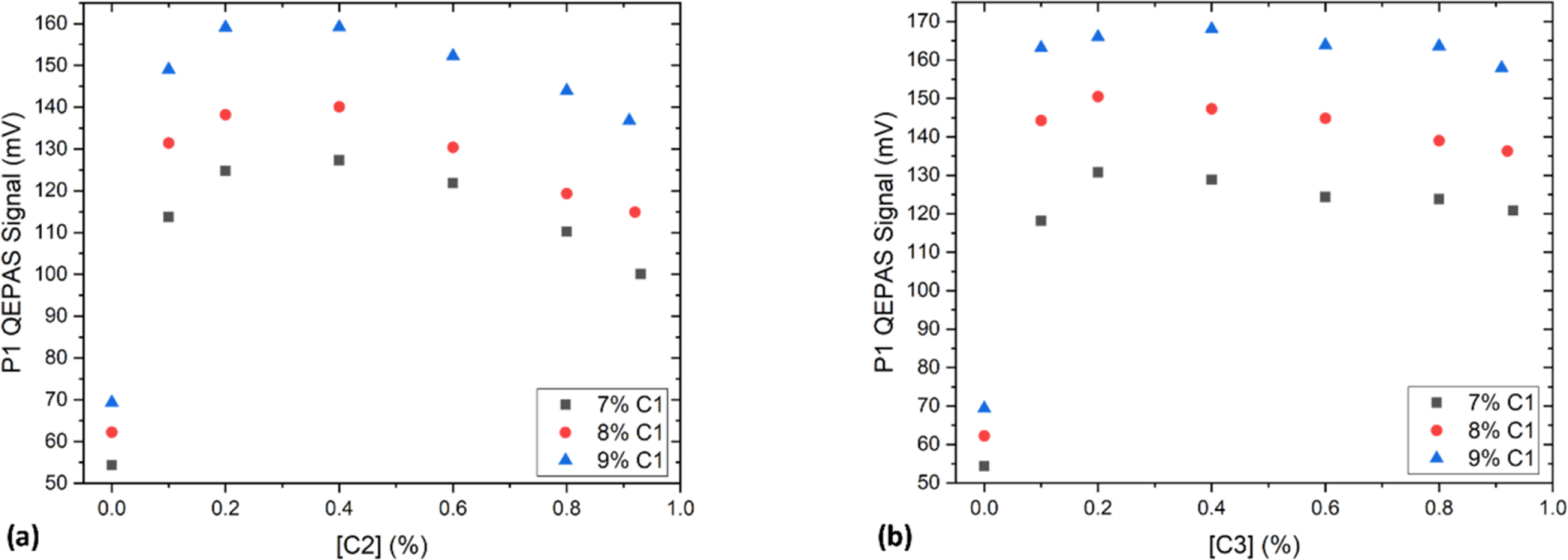
P1 peak QEPAS signal for a fixed concentration of methane ranging from 7.00 to 9.00% when varying the (a) C2 and (b) C3 concentrations within two gas mixtures.

As reported in the literature, in the explored concentration range, the photoacoustic signal generated by the C1 absorption within a NG-like mixture is affected by the matrix composition.^19^ For this reason, the influence of C2 and C3 concentration variations on the C1 photoacoustic signal was investigated. This analysis was carried out by generating several C1–C2 and C1–C3 mixtures and acquiring the QEPAS signal on the P1 peak. The dependence of the P1 QEPAS signal on C2 and C3 concentrations is shown in Figure 4a,b, varying the C1 concentration in the 7.00–9.00% range.

First, by looking at the trend in Figure 4a, a significant influence of C2 on the P1 values in the different mixtures can be inferred. Indeed, the promoting action of C2 on the C1 relaxation leads to a sharp increase by a factor of 2 up to 0.2% C2. In contrast to the saturation in the C1 signal previously observed,^19^ the decrease in the P1 signal for concentrations higher than 0.6% (Figure 4a) is due to the spectral interference with the negative lobe of the C2 absorption peak at 2966.17 cm^–1^ (see Figure 2).^37^ The dependence of the C1 signal on the C3 concentration is reported in Figure 4b. In this case, a sharp increase (a factor of ∼2.4) in the P1 signal is observed for C3 concentrations up to 0.2%, with a roll-off for C3 concentrations higher than 0.4%. This behavior has already been predicted and observed for other gases, e.g., water vapor mixed with C1.^30,32^ The influence of different C3 concentrations on P2 in two gas mixtures was investigated as well; however, no mutual effects on the measured QEPAS signals were observed. Therefore, both spectral interferences and matrix effects were observed in the analyzed gas samples, thus requiring a suitable analysis tool capable of filtering them out.

## 4 Partial Least-Squares Regression in Gas Spectroscopy: Fundamentals

PLSR is a multivariate analysis (MVA) technique, commonly employed to handle noise, missing values, and correlations among nominally independent variables belonging to relatively small datasets.^38^ Recently, PLSR has been effectively employed as an analysis tool for filtering out mutual spectral interferences and matrix effects in PAS.^19,30,34,40^ This technique is based on a simple regression model, which reads in matrix form:
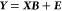
2where ***X*** is the predictor matrix, ***Y*** is the response matrix, ***B*** is the regression coefficient matrix, and ***E*** is the residual (or error) matrix.^39^ In optical spectroscopy, the rows of the ***X*** matrix consist of the acquired spectra, while the nominal concentrations of the mixtures are the rows of the ***Y*** matrix. During the training step of the algorithm,^19,32^ the regression coefficient matrix is retrieved by maximizing the covariance matrix cov(***X***,***Y***), with the only assumption that the analyzed system can be described in terms of a small subset of truly independent variables or “latent variables” (LVs).^39,41^ From this point of view, PLSR involves the projection to a latent structure of the original system, meaning that the analysis is performed in a subspace of the original wavelength space with a reduced dimensionality, given by the number of LVs.^42^ The assessment of the optimal LV number is crucial to avoid possible under- or overfittings of the model, and it is typically carried out by employing two figures of merit: the explained variance maximization and the cross-validation (CV) algorithm.^43^

One of the main advantages of the PLSR approach, when applied to PAS, is the possibility of accurately retrieving the concentration of each analyte within a complex gas mixture by only analyzing the cross-correlations among different wavelengths of the same spectrum. Indeed, there is no need for an a priori knowledge of the photoacoustic relaxation cascade dynamics, contrarily to other methods relying on the calculation of the full relaxation cascade, which may lose their effectiveness when the matrix complexity increases.^30−32,40^

## 5 PLSR Analysis: Algorithm Training and Test

The input data set of the PLSR algorithm was built acquiring 71 spectra with different sample compositions, including 2- and 3-gas mixtures (i.e., C1–C2, C1–C3, C2–C3, and C1–C2–C3) in nitrogen. The concentration ranges considered for the data analysis process are 7.0–9.0% for C1 and 0.1–1.0% for C2 and C3. Each spectrum includes 346 data points, with a spectral sampling of ∼0.0095 cm^–1^ (Figure 2).

**Figure 5:**
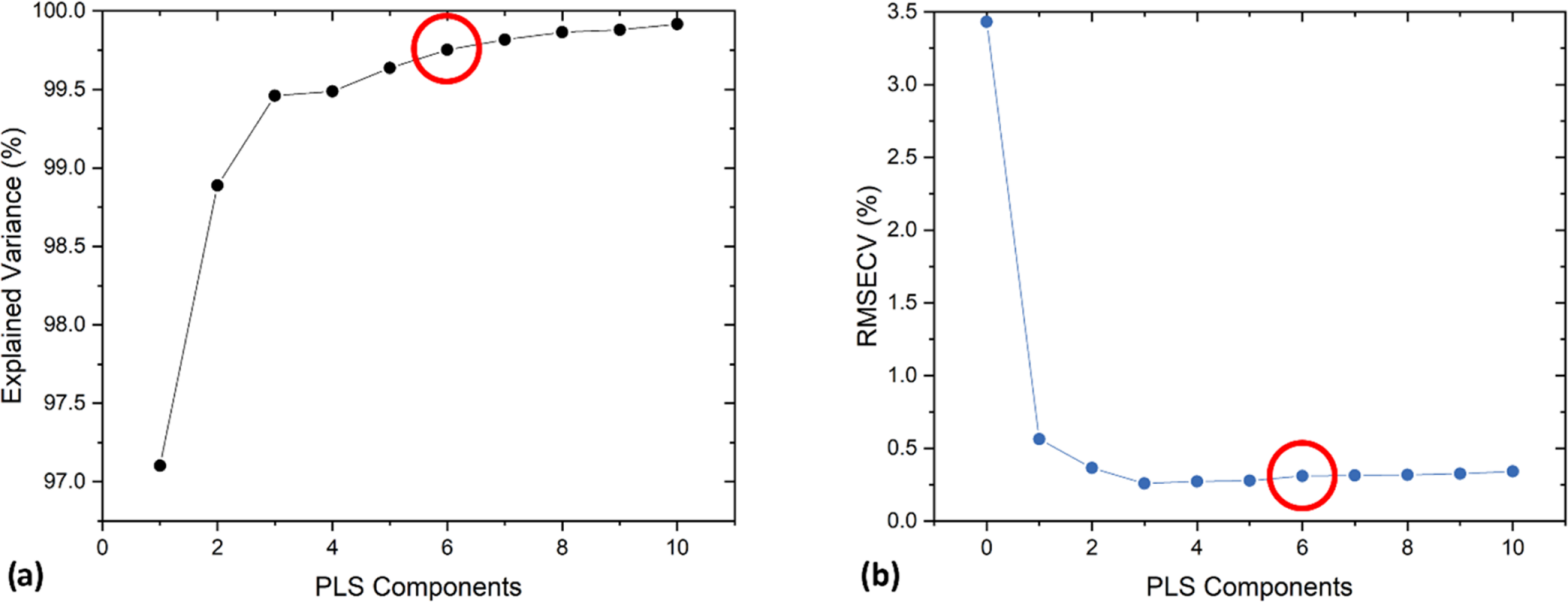
(a) Explained variance and (b) root-mean-square error of cross validation (RMSECV) evaluated for each LV (or PLS component) up to 10 employing the full input dataset. The selected number of LVs is 6, as highlighted by the red circle.

A 10-fold cross-validation (CV) algorithm was applied to the full data set to determine the optimal number of LVs for the subsequent PLS analysis. Both the explained variance and the root-mean-square error of cross validation (RMSECV) were evaluated for each LV, as shown in Figure 5a,b, respectively.

As it can be noticed, the RMSECV is minimized for three LVs, remaining substantially constant for a higher number of PLS components. At the same time, the explained variance is first maximized for three LVs, although it increases significantly up to six LVs, which was selected as the optimal number of components for analysis. Indeed, a higher number of PLS components would lead to only negligible increments. Even though the entire analysis process does not provide a physical interpretation of the LVs, it is still possible to interpret them in a qualitative way as independent contributions to the QEPAS spectra. Namely, the first three of them may be considered as the independent contributions of the three analytes composing the mixtures; the others may be considered as the matrix effects related to (i) the photoacoustic relaxation of C1 through C2, (ii) the photoacoustic relaxation of C1 through C3, and (iii) the self-relaxation of each analyte, which may be assumed to become relevant in the explored concentration ranges.^19^

**Figure 6:**
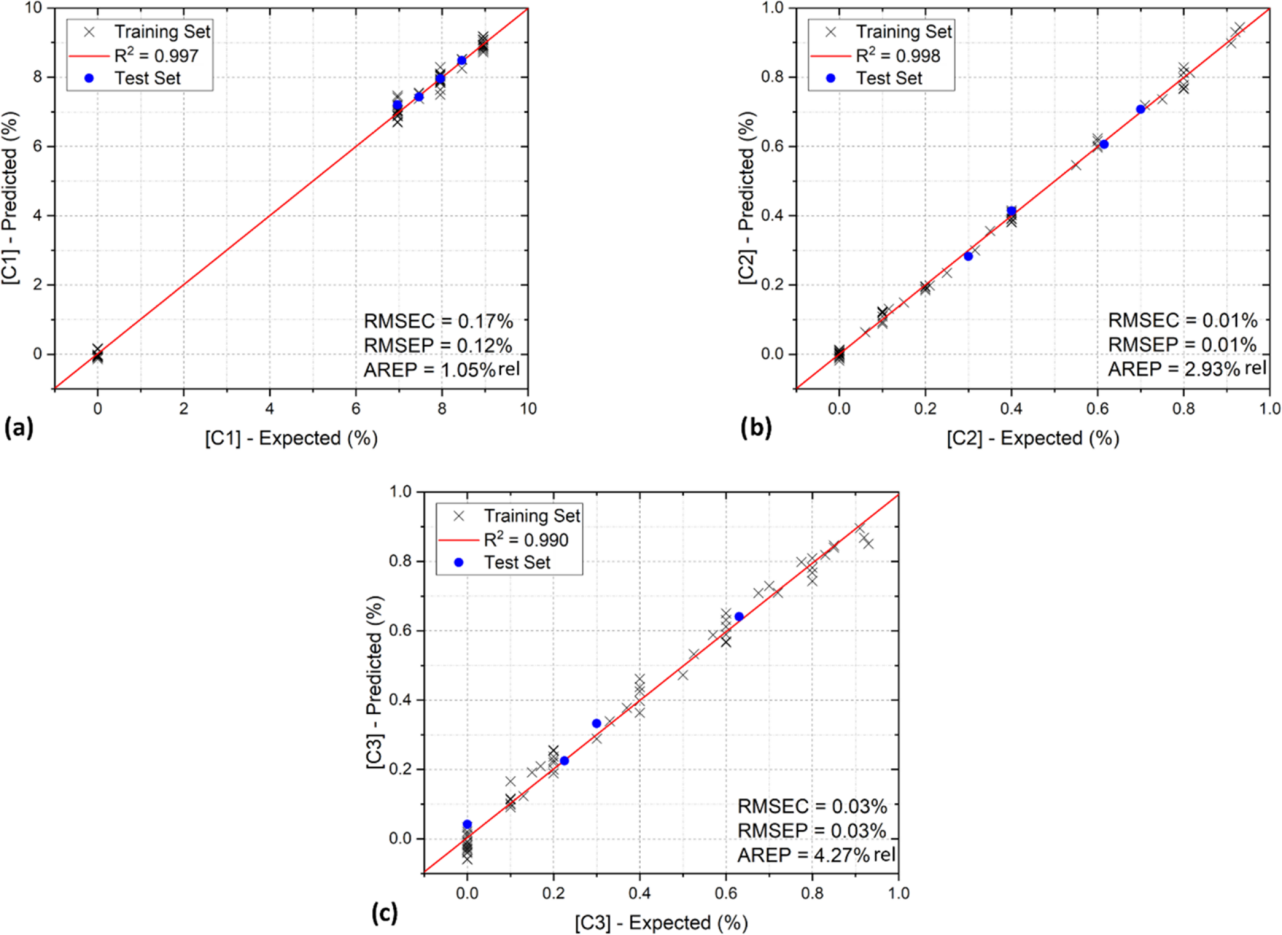
Outcome of the PLSR algorithm for (a) C1; (b) C2; and (c) C3 for a representative test set. For each analyte, the RMSEC, the RMSEP, and the AREP are displayed.

The PLSR analysis was carried out by means of a MATLAB-based algorithm, splitting the acquired dataset into a training set and a test set. The two sets are then employed to calibrate and test the model, respectively. The coefficient matrix retrieved from the calibration step (see eq 2) is employed to predict the C1, C2, and C3 concentrations of the corresponding test set. Five independent test sets were assembled, each one consisting of four spectra randomly picked from the full dataset. For each test set, the root-mean-square error of calibration (RMSEC, %) and root mean square error of prediction (RMSEP, %) were evaluated together with the average relative error of prediction (AREP, % rel). The RMSEC parameter provides quantitative information about the algorithm calibration, thus returning information on its precision, while the RMSEP parameter provides information about the discrepancy between the expected values and the obtained results, thus returning information about its accuracy.^44^ The results of the calibration and the test step of the PLSR analysis for a representative test set are shown in Figure 6a–c for C1, C2, and C3, respectively. The expected and predicted concentrations calculated for each test set are reported in Table 2, together with their uncertainties. The uncertainty on the expected concentration has been evaluated starting from the expanded uncertainty on the gas cylinders and the relative uncertainty on the dilution (1%), while the RMSEC has been adopted as the uncertainty on the predicted concentrations.

**Table 2:** Predicted and Expected Concentrations for Each Analyte and Each Test Set

	C1		C2		C3	
	expected (%)	predicted (%)	expected (%)	predicted (%)	expected (%)	predicted (%)
test set #1	7.96 ± 0.18	7.96 ± 0.17	0.40 ± 0.01	0.41 ± 0.01	0	0.04 ± 0.03
	6.97 ± 0.16	7.21 ± 0.17	0.30 ± 0.01	0.28 ± 0.01	0.63 ± 0.01	0.64 ± 0.03
	7.46 ± 0.17	7.43 ± 0.17	0.70 ± 0.02	0.71 ± 0.01	0.23 ± 0.01	0.22 ± 0.03
	8.46 ± 0.19	8.48 ± 0.17	0.62 ± 0.01	0.61 ± 0.01	0.30 ± 0.01	0.33 ± 0.03
test set #2	7.96 ± 0.18	7.85 ± 0.16	0.20 ± 0.01	0.18 ± 0.01	0	0.03 ± 0.03
	6.97 ± 0.16	7.28 ± 0.16	0.10 ± 0.01	0.11 ± 0.01	0.83 ± 0.02	0.80 ± 0.03
	7.46 ± 0.17	7.74 ± 0.16	0.40 ± 0.01	0.38 ± 0.01	0.53 ± 0.01	0.51 ± 0.03
	8.96 ± 0.20	9.47 ± 0.16	0.71 ± 0.02	0.72 ± 0.01	0.20 ± 0.01	0.26 ± 0.03
test set #3	7.46 ± 0.17	7.36 ± 0.17	0.25 ± 0.01	0.23 ± 0.01	0.68 ± 0.02	0.69 ± 0.03
	7.96 ± 0.18	7.93 ± 0.17	0.55 ± 0.01	0.54 ± 0.01	0.37 ± 0.01	0.37 ± 0.03
	8.46 ± 0.19	8.02 ± 0.17	0.82 ± 0.02	0.81 ± 0.01	0.10 ± 0.01	0.08 ± 0.03
	8.96 ± 0.20	8.92 ± 0.17	0.06 ± 0.01	0.06 ± 0.01	0.85 ± 0.02	0.83 ± 0.03
test set #4	6.97 ± 0.16	6.69 ± 0.16	0.93 ± 0.02	0.94 ± 0.01	0	–0.05 ± 0.03
	7.46 ± 0.17	7.57 ± 0.16	0.40 ± 0.01	0.38 ± 0.01	0.53 ± 0.01	0.52 ± 0.03
	7.96 ± 0.18	7.95 ± 0.16	0.75 ± 0.02	0.74 ± 0.01	0.17 ± 0.01	0.21 ± 0.03
	8.96 ± 0.20	8.77 ± 0.16	0.21 ± 0.01	0.20 ± 0.01	0.70 ± 0.02	0.73 ± 0.03
test set #5	8.96 ± 0.20	9.14 ± 0.16	0.60 ± 0.01	0.60 ± 0.01	0	–0.01 ± 0.03
	6.97 ± 0.16	6.87 ± 0.16	0.80 ± 0.02	0.80 ± 0.01	0.13 ± 0.01	0.13 ± 0.03
	7.96 ± 0.18	7.64 ± 0.16	0.20 ± 0.01	0.19 ± 0.01	0.72 ± 0.02	0.69 ± 0.03
	8.46 ± 0.19	8.62 ± 0.16	0.12 ± 0.01	0.14 ± 0.01	0.80 ± 0.02	0.77 ± 0.03

The data collected as the outcome of the algorithm display a clear linear trend for each analyte, and the superimposed linear fits always fall close to the bisector of the plane, as expected, with an *R*^2^ value always higher than 0.990. Furthermore, the significant decrease in the relative prediction accuracy observed on C3 with respect to the other two analytes can be attributed to the lower number of C3 absorption features within the targeted spectral range as well as to the lower signal-to-noise ratio (SNR) of the C3 QEPAS signals, as can be noticed from the acquired spectra (e.g., Figure 2). Therefore, it can be inferred that a low SNR induces a degradation of the PLSR algorithm prediction accuracy with respect to one or more analytes composing the gas mixture.

From Table 2, it can be noticed that the RMSEC varies between 0.16% and 0.17% for C1 and 0.01% and 0.02% for C2 and C3 when changing the test set removed from the full dataset. This proves that both the calibration and the validation steps are unbiased. Furthermore, the predicted and expected concentrations are compatible within their uncertainties (1σ), with only a few minor exceptions.

The mean RMSEC, RMSEP, and AREP evaluated over all five test sets are reported in Table 3. The average AREP values point out an algorithm accuracy of ∼98%, ∼96%, and ∼93% for C1, C2, and C3 concentration predictions, respectively. It is worth noting that varying the number of LVs in a neighborhood of 6 does not lead to substantial variations of the average AREP values, hence confirming the validity of the choice.

**Table 3:** RMSEC, RMSEP, and AREP Averaged Out of All of the Test Sets for Each Analyte

	C1	C2	C3
mean RMSEC (%)	0.16	0.01	0.03
mean RMSEP (%)	0.22	0.01	0.03
mean AREP (% rel)	2.19	4.31	6.50

## 6 Conclusions

In this work, a compact and portable QEPAS sensor for in situ and real-time detection of light alkanes, namely, C1, C2, and C3, in natural gas-like mixtures is presented. The sensor consists of a 19″-diagonal aluminum rack box, enclosing the sensor head, the electronics, and the gas handling system. Natural gas component analysis has been carried out in the laboratory generating mixtures of C1 (7.00–9.00%), C2 (0–1.00%), and C3 (0–1.00%) in nitrogen, starting from certified cylinders. PLSR analysis has been implemented by means of a MATLAB-based code to filter out both spectral interferences and matrix effects observed in the acquired QEPAS spectra, thus allowing the retrieval of C1, C2, and C3 concentrations with an accuracy of ∼98%, ∼96%, and ∼93%, respectively. These results mark a significant improvement with respect to the previously reported demonstrations of QEPAS sensors for natural gas analysis from the point of view of^19,23^ (i) the applicability of the multivariate approach to complex and highly variable NG gas matrices, (ii) the prediction accuracy, and (ii) the capability to detect and discriminate C3 within natural gas-like complex mixtures.

Further developments of this technology will be focused on field testing the sensor prototype and extending the detection of the natural gas components to the heavier alkanes, i.e., C4, C5, and C6, as well as to the nonhydrocarbon components, e.g., carbon dioxide and hydrogen sulfide. Indeed, these features would translate into more accurate, continuous, and reliable assessment of the most relevant gases at various stages in oil and gas operations, from exploration, drilling, and production to hydrocarbon processing and distribution.

## Abbreviations

ADM: acoustic detection module
AR: acoustic resonator
C1: methane
C2: ethane
C3: propane
C4: butane
C5: pentane
FID: flame ionization detector
GC: gas chromatograph
GHG: greenhouse gas
MS: mass spectrometry
NG: natural gas
MVA: multivariate analysis
PLSR: partial least-squares regression
QEPAS: quartz-enhanced photoacoustic spectroscopy
QTF: quartz tuning fork
SMR: steam methane reforming
